# Long-term outcomes of high-risk HR-positive and HER2-negative early breast cancer patients from GEICAM adjuvant studies and El Álamo IV registry

**DOI:** 10.1007/s10549-023-07002-1

**Published:** 2023-06-20

**Authors:** Miguel Martín, Eva Carrasco, Álvaro Rodríguez-Lescure, Raquel Andrés, Sonia Servitja, Antonio Antón, Manuel Ruiz-Borrego, Begoña Bermejo, Ángel Guerrero, Manuel Ramos, Ana Santaballa, Montserrat Muñoz, Josefina Cruz, Sara Lopez-Tarruella, Jose I. Chacón, Isabel Álvarez, Purificación Martínez, Juan J. Miralles, Óscar Polonio, Carlos Jara, David Aguiar-Bujanda

**Affiliations:** 1 Hospital Universitario Gregorio Marañón, Instituto de Investigación Sanitaria Gregorio Marañón (IiSGM), Universidad Complutense, Madrid, Spain; 2 GEICAM, Spanish Breast Cancer Group, Madrid, Spain; 3 Centro de Investigación Biomédica en Red de Oncología, CIBERONC-ISCIII, Madrid, Spain; 4 Hospital General Universitario de Elche, Elche, Spain; 5 Hospital Clínico Universitario Lozano Blesa, Zaragoza, Spain; 6 Hospital del Mar, Barcelona, Spain; 7 Hospital Universitario Miguel Servet, Universidad de Zaragoza, Instituto Investigación Sanitaria Aragón (IISA), Zaragoza, Spain; 8 Hospital Universitario Virgen del Rocío, Sevilla, Spain; 9 Hospital Clínico Universitario de Valencia, Biomedical Research Institute INCLIVA. Universidad de Valencia, Valencia, Spain; 10 Instituto Valenciano de Oncología, Valencia, Spain; 11 Centro Oncológico de Galicia, A Coruña, Spain; 12 Hospital Universitario La Fe, Valencia, Spain; 13 Department of Medical Oncology and Translational Genomics and Targeted Therapies in Solid Tumors, IDIBAPS, Barcelona, Spain; 14 Hospital Universitario de Canarias, Santa Cruz de Tenerife, Spain; 15 Hospital Universitario de Toledo, Toledo, Spain; 16 Hospital de Donostia, San Sebastian, Spain; 17 Hospital Universitario Basurto, Bilbao, Spain; 18 Hospital Universitario Fundación Alcorcón, Alcorcón, Spain; 19 Hospital Universitario de Gran Canaria Dr. Negrín, Las Palmas de Gran Canaria, Spain

**Keywords:** High-risk, Early breast cancer, Hormone receptor (HR)-positive, HER2-negative

## Abstract

**Purpose:**

The monarchE trial showed that the addition of abemaciclib improves efficacy in patients with high-risk early breast cancer (EBC). We analyzed the long-term outcomes of a population similar to the monarchE trial to put into context the potential benefit of abemaciclib.

**Methods:**

HR-positive/HER2-negative EBC patients eligible for the monarchE study were selected from 3 adjuvant clinical trials and a breast cancer registry. Patients with ≥ 4 positive axillary lymph nodes (N +) or 1–3 N + with tumor size ≥ 5 cm and/or histologic grade 3 and/or Ki67 ≥ 20%, who had undergone surgery with curative intent and had received anthracyclines ± taxanes and endocrine therapy in the neoadjuvant and /or adjuvant setting were included. We performed analysis of Invasive Disease-Free Survival (iDFS), Distant Disease-Free Survival (dDFS) and Overall Survival (OS) at 5 and 10 years, as well as yearly (up to 10) of Invasive Relapse Rate (IRR), Distant Relapse Rate (DRR) and Death Rate (DR).

**Results:**

A total of 1,617 patients were analyzed from the GEICAM-9906 (312), GEICAM-2003–10 (210), and GEICAM-2006–10 (160) trials plus 935 from *El Álamo IV*. With a median follow-up of 10.1 years, the 5 and 10 years iDFS rates were 75.2% and 57.0%, respectively. The dDFS and OS rates at 5 years were 77.4% and 88.8% and the respective figures at 10 years were 59.7% and 70.9%.

**Conclusions:**

This data points out the need for new therapies for those patients. A longer follow-up of the monarchE study to see the real final benefit with abemaciclib is warranted.

**Trial registration:**

ClinTrials.gov: GEICAM/9906: NCT00129922; GEICAM/ 2003-10: NCT00129935 and GEICAM/ 2006-10: NCT00543127.

**Supplementary Information:**

The online version contains supplementary material available at 10.1007/s10549-023-07002-1.

## Introduction

Chemotherapy followed by endocrine therapy (ET) is the current (neo)adjuvant standard of care for high-risk hormone receptor (HR)-positive and human epidermal growth factor receptor 2 (HER2)-negative early breast cancer (EBC) patients [1].

The monarchE study [2] is an open-label, randomized, phase 3 clinical trial investigating the addition of abemaciclib, a cyclin-dependent kinase (CDK) 4 and 6 inhibitor to standard ET (after chemotherapy, surgery, and radiotherapy) in patients with high-risk HR-positive and HER2-negative breast cancer. This study included 5,637 patients with high-risk of relapse characteristics defined as the presence of at least 4 positive axillary lymph nodes (N +) or 1–3 N + plus one or more of the following features: tumor size of at least 5 cm, histologic grade 3 and/or high centrally determined Ki67 (≥ 20%). At the last follow-up analysis reported so far, with 42 months median follow-up and 99.2% of patients off treatment [3], the 4-year Invasive Disease-Free Survival (iDFS) rate in the abemaciclib + ET arm was 85.8% versus 79.4% in the ET alone arm with an absolute improvement of 6.4% (HR: 0.664, p < 0.0001). The 4-year Distant Relapse-Free Survival (dRFS) rates also showed an absolute improvement of 5.9% in favor of abemaciclib (88.4% in the experimental arm and 82.5% in the control arm).

This important benefit was already observed very early at the interim analysis with a median follow-up of 15.5 months [2], which is unusual in studies with endocrine agents, and has been increasing with further follow-up [4, 5]. Adjuvant studies and registries with long follow-up can provide relevant information regarding the expected long-term outcomes of high-risk patients and could be useful to better understand the potential overall long-term benefit with abemaciclib.

## Methods

The analyzed cohort includes data from 3 adjuvant randomized clinical trials in EBC patients and one breast cancer (BC) registry. In the GEICAM/9906 [6], patients with N + received six cycles of fluorouracil/epirubicin/cyclophosphamide (FEC) versus four cycles of FEC followed by eight administrations of weekly paclitaxel. In the GEICAM/2003-10 [7], patients with N + received four cycles of epirubicin/cyclophosphamide followed by 4 cycles of docetaxel versus four cycles of epirubicin/docetaxel followed by 4 cycles of capecitabine. In these two studies, women with HR-positive tumors were scheduled to receive 5 years of ET after the end of chemotherapy (tamoxifen or aromatase inhibitors, or a sequence of both). In the GEICAM/2006-10 [8], patients received anastrozole (for 5 years) versus anastrozole (for 5 years) combined with fulvestrant (for the first 3 years). These studies included patients diagnosed from 1999 to 2010. *El Álamo IV* is a retrospective registry performed to characterize BC cases diagnosed in Spain between 2002 and 2005 in 43 Spanish sites.

The current study was approved by an institutional review board. The data was analyzed at the Statistical Unit of GEICAM using SAS, version 9.4 (SAS Institute Inc.).

### Patients

For this analysis we included women and men with similar characteristics to those included in the monarchE study from the previously described studies. Patients should have HR-positive and HER2-negative EBC, with high-risk of relapse defined as the presence of at least 4 N + , or of 1–3 N + plus either tumor size ≥ 5 cm and/or histologic grade 3 and/or Ki67 levels ≥ 20%. Patients should have undergone surgery with curative intent and received neoadjuvant and/or adjuvant chemotherapy with anthracyclines with or without taxanes and ET.

### Objectives and endpoints

The objectives were to determine iDFS, Distant Disease-Free Survival (dDFS) and Overall Survival (OS) at 5 and 10 years. Other objectives were the yearly (in years 1 to 10) evaluation of Invasive Relapse Rate (yIRR), Distant Relapse Rate (yDRR) and Death Rate (yDR). iDFS was defined as time from adjuvant ET initiation to the first date of diagnosis of any of the following events: ipsilateral BC relapse, local/regional BC relapse, distant BC relapse, contralateral invasive BC, second primary invasive cancer non-BC and death due to any cause. yIRR was defined as the proportion of patients with any of the following events: ipsilateral BC relapse, local/regional BC relapse or distant BC relapse per year. dDFS was defined as time from adjuvant ET initiation to the first date of diagnosis of any of the following events: distant BC relapse, second primary invasive cancer non-BC and death due to any cause. yDRR was defined as the proportion of patients with distant BC relapse per year (either as first or subsequent relapse if first relapse was local/regional). yDR was defined as the proportion of deaths per year. OS was defined as time from adjuvant ET initiation to the date of death from any cause.

### Statistical analysis

The Kaplan–Meier limit-product method was used to estimate time to event endpoints (iDFS, dDFS and OS) and the survival curves were presented graphically. Yearly time to events with the 95% confidence interval (CI) were reported.

The Hazard function, which is the mean relative event incidence rate, was used to evaluate the yIRR, yDRR and yDR in years 1 to 10.

All statistical tests used in the analysis were two-sided and calculated with a significance level of 0.05.

## Results

### Patients characteristics

**Fig. 1:**
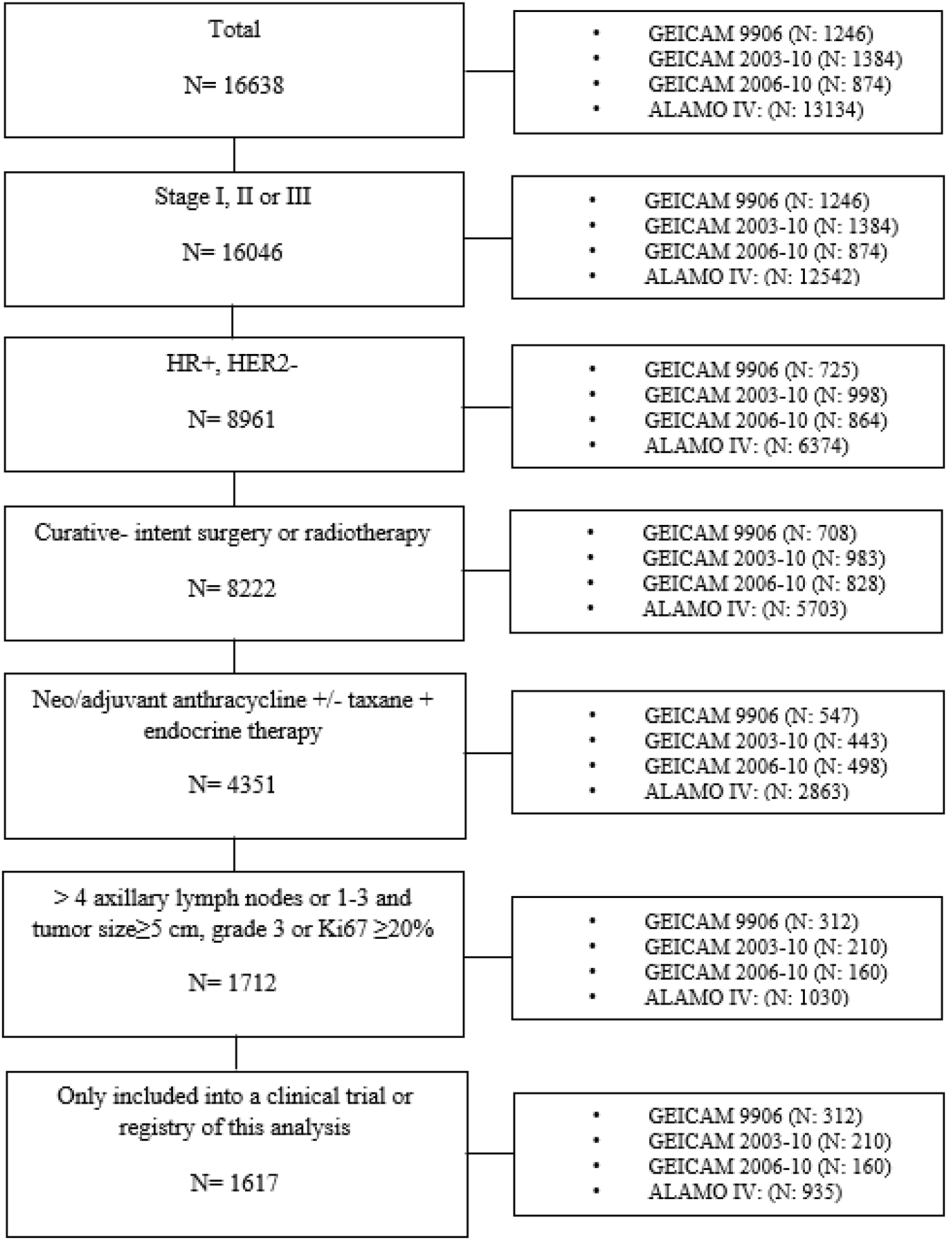
Consort diagram

We selected 1,617 patients for the analysis, 312 from the GEICAM/9906, 210 from the GEICAM/2003-10, 160 from the GEICAM/ 2006-10 and 935 from *El Álamo IV* registry (Fig. 1). The latter represents 16.2% of all HR-positive and HER2-negative EBC patients included in *El Álamo IV* registry.

Baseline demographic and disease characteristics are described in Table 1. Median age was 54 years; 0.7% were male and 34.9% and 62.1% were pre- and postmenopausal women, respectively; 44.1% had grade 3 tumors and 39.2% had tumors with Ki67 ≥ 20%. Taxane-based chemotherapy was received by 66.9% of patients and most of the remaining patients received anthracycline combinations without taxanes. Most patients (80.7%) received chemotherapy only in the adjuvant setting; neoadjuvant therapy was received by 19.3% of patients (10.8% in the neoadjuvant setting only and 8.5% in both). The median exposure time to adjuvant ET was 5 years (range: 0.04–15.03 years), with 8.7% and 1.8% of patients receiving more than 7 and more than 10 years of ET, respectively. Aromatase inhibitors were administered, either alone, in combination or in sequence in 71.9% of patients. LHRH analogues were administered to 15.1% of premenopausal patients.

**Table 1:** Baseline characteristics of patients

	Total
	N = 1617
Median age, years (range)	54 (23–89)
Menopausal status, n (%)	
Postmenopausal	1002 (62.0%)
Premenopausal	566 (35.0%)
Unknown	38 (2.4%)
Males	11 (0.7%)
Histologic type, n (%)	
Invasive Ductal Carcinoma	1311 (81.1%)
Invasive Lobular Carcinoma	244 (15.1%)
Other	61 (3.8%)
Stage, n (%)	
I*	2 (0.1%)
IIA	230 (14.2%)
IIB	269 (16.6%)
IIIA	714 (44.2%)
IIIB	114 (7.1%)
IIIC	288 (17.8%)
Number of positive axillary lymph nodes, n (%) +	
1–3	601 (37.2%)
≥ 4	1016 (62.8%)
Histopathological grade, n (%)	
G1, well differentiated	165 (10.9%)
G2, moderately differentiated	579 (38.2%)
G3, poorly differentiated	668 (44.1%)
GX, unknown / missing	104 (6.9%)
Ki67 level, n (%)	
< 20%	329 (56.0%)
≥ 20%	230 (39.2%)
Not classifiable	28 (4.8%)
Breast Surgery, n (%)	
Conservative	681 (42.1%)
Mastectomy	936 (57.9%)
Axillary Surgery, n (%)	
Full ALND	1440 (89.1%)
SLND	13 (0.8%)
SLND followed by full ALND	151 (9.3%)
Non surgery/ Not determined	13 (0.8%)
Chemotherapy setting	
Adjuvant	1305 (80.7%)
Neoadjuvant	174 (10.8%)
Neoadjuvant + Adjuvant	138 (8.5%)
Type of Chemotherapy	
Anthracycline	536 (33.1%)
Anthracycline + Taxane	1081 (66.9%)
Type of Endocrine Therapy†	
SERM + Aromatase Inhibitor	626 (38.7%)
SERM	449 (27.8%)
Aromatase Inhibitor	454 (28.1%)
Aromatase Inhibitor + SERD	83 (5.1%)
Other	5 (0.3%)
Duration of ET	
Up to 5 years	886 (54.8%)
5–7 years	561 (34.7%)
7–10 years	141 (8.7%)
> 10 years	29 (1.8%)

^*^Patients receiving neoadjuvant treatment were classified using their cTNM; however, all selected patients had pathologic positive nodes

+ In patients treated with neoadjuvant therapy pN was used to report the number of positive axillary nodes. In 12 patients, the axillary surgery information was not available; for them we used the cN information with the assumption that cN1 was corresponding to 1–3 nodes and cN2-3 to ≥ 4 nodes

^†^LHRH analogues were administered to 15.1% of premenopausal patients (9.2% in combination with SERMs, 4.8% in combination with SERMs and aromatase inhibitors [combined or in sequence] and 1.1% in combination with aromatase inhibitors). The use of aromatase inhibitors versus SERMs was not different according to the histologic type (aromatase inhibitors were administered to 71.2% of patients with ductal tumors versus 73.8% with lobular tumors)

ALND denotes Axillary Lymph Nodes Dissection, SLND denotes Sentinel Lymph Nodes Dissection, SERM denotes Selective Estrogen Receptor Modulator and SERD denotes Selective Estrogen Receptor Degrader

### Efficacy

**Fig. 2:**
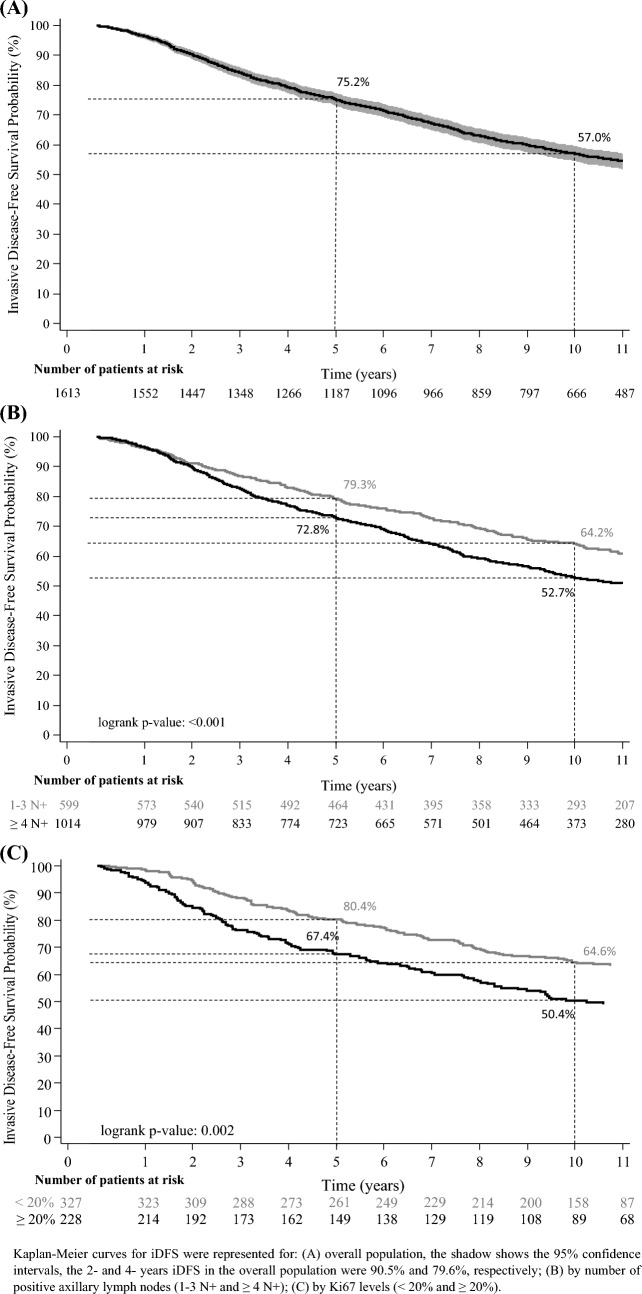
Invasive Disease-free Survival (iDFS) from the start of adjuvant ET. A. Overall population; B. By number of positive axillary lymph nodes (1–3 N + and ≥ 4 N +); C. By Ki67 levels (< 20 and ≥ 20%). Kaplan–Meier curves for iDFS were represented for: (A) overall population, the shadow shows the 95% confidence intervals, the 2- and 4- years iDFS in the overall population were 90.5 and 79.6%, respectively; (B) by number of positive axillary lymph nodes (1–3 N + and ≥ 4 N +); (C) by Ki67 levels (< 20 and ≥ 20%)

**Fig. 3:**
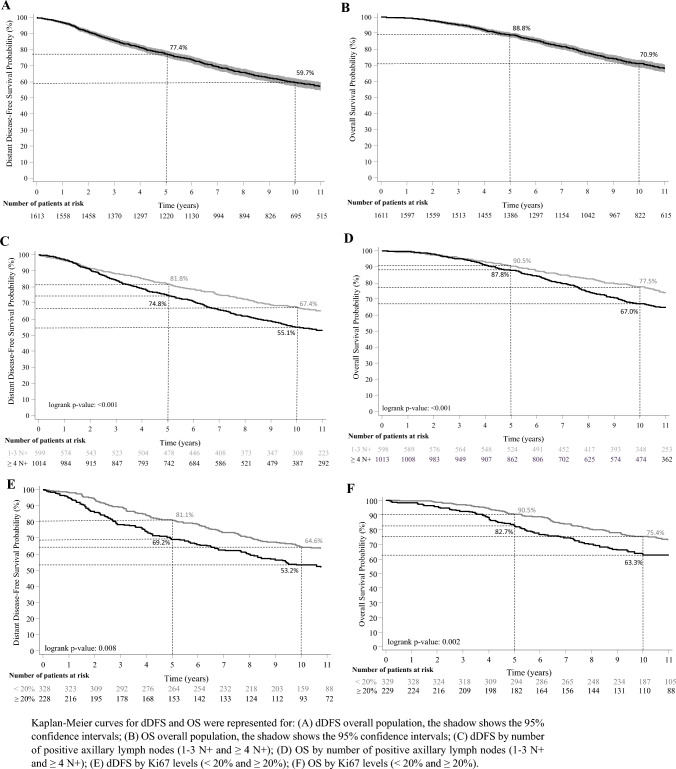
Distant Disease-free Survival (dDFS) and Overall Survival (OS) from the start of adjuvant ET. A. dDFS overall population; B. OS overall population C. dDFS by number of positive axillary lymph nodes (1–3 N + and ≥ 4 N +); D. OS by number of positive axillary lymph nodes (1–3 N + and ≥ 4 N +); E. dDFS by Ki67 levels (< 20 and ≥ 20%); F. OS by Ki67 levels (< 20 and ≥ 20%). Kaplan–Meier curves for dDFS and OS were represented for: (A) dDFS overall population, the shadow shows the 95% confidence intervals; (B) OS overall population, the shadow shows the 95% confidence intervals; (C) dDFS by number of positive axillary lymph nodes (1–3 N + and ≥ 4 N +); (D) OS by number of positive axillary lymph nodes (1–3 N + and ≥ 4 N +); (E) dDFS by Ki67 levels (< 20 and ≥ 20%); (F) OS by Ki67 levels (< 20 and ≥ 20%)

With a median follow-up time of 10.1 years (range 0.2–18 years), the 5 and 10 years iDFS rates were 75.2 and 57.0%, respectively, see Fig. 2A and supplementary Table 1 (for information about the specific events). At 5 and 10 years, the dDFS rates were 77.4 and 59.7% (Fig. 3A and supplementary Table 1) and the OS rates were 88.8 and 70.9%, respectively (Fig. 3B).

We performed subgroups analyses by number of nodes (1–3 N + and ≥ 4 N +) and Ki67 levels (< 20 and ≥ 20%). Outcomes at 5 and 10 years in patients with 1–3 N + were the following: iDFS rates of 79.3 and 64.2% (Fig. 2B), dDFS rates of 81.8 and 67.4% (Fig. 3C) and OS rates of 90.5 and 77.5% (Fig. 3D); the respective figures for patients with ≥ 4 N + were 72.8 and 52.7% for iDFS (Fig. 2B), 74.8 and 55.1% for dDFS (Fig. 3C) and 87.8 and 67.0% for OS (Fig. 3D). Outcomes at 5 and 10 years in patients with Ki67 < 20% were the following: iDFS rates of 80.4% and 64.6% (Fig. 2C), dDFS rates of 81.1% and 64.6% (Fig. 3E) and OS rates of 90.5% and 75.4% (Fig. 3F); the respective figures for patients with Ki67 ≥ 20%, were 67.4% and 50.4% for iDFS (Fig. 2C), 69.2 and 53.2% for dDFS (Fig. 3E) and 82.7 and 63.3% for OS (Fig. 3F).

**Fig. 4:**
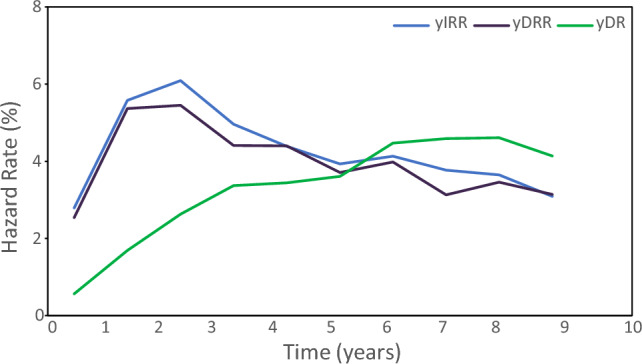
Yearly Invasive Recurrence rate (yIRR), Yearly Distant Recurrence Rate (yDRR) and Yearly Death Rate (yDR) in years 1 to 10. The figure shows the evolution of yIRR, yDRR and yDR in years 1 to 10 in the overall population

The yIRR, yDRR and yDR in years 1 to 10 are shown in supplementary Table 2 and represented graphically in Fig. 4. The cumulative IRR events at 5 and 10 years were 21.44% and 33.33%, respectively and yIRR increased from year 2, till year 3 followed by a second peak at years 7–8. The cumulative DRR events at 5 and 10 years were 20.07 and 31.41%, respectively. Similar to what was seen with yIRR, yDRR increased from year 2, till year 3 followed by a second peak at years 7–8. The cumulative DR events at 5 and 10 years were 11.05 and 26.51%, respectively and yDR increased steadily from year 1 to year 10.

We also performed subgroup exploratory analyses by type of ET regimen (selective estrogen receptor modulator [SERM]s plus aromatase inhibitors, SERMs and aromatase inhibitors). The 5 and 10 years iDFS rates in patients who received SERMs plus aromatase inhibitors were 87.8% and 69.2%, in patients treated with SERMs were 55.2% and 41.1% and in patients treated with aromatase inhibitors were 78.2% and 57.1% (supplementary Fig. 1A, 1B and 1C). The 5 and 10 years dDFS rates in patients who received SERMs plus aromatase inhibitors were 89.4% and 71.1%, in patients treated with SERMs were 58.1% and 44.9% and in patients treated with aromatase inhibitor were 80.8% and 59.7% (supplementary Fig. 2A, B and C). OS rates in patients who received SERM plus aromatase inhibitor were 94.4% and 81.1%, in patients treated with SERM were 80.3% and 55.0% and in patients treated with aromatase inhibitor were 90.4% and 73.8% (supplementary Fig. 3A, B and C).

## Discussion

Patients with operable breast cancer considered at high-risk of relapse have a dismal prognosis. The definition of high-risk is relatively subjective, since the prognosis of breast cancer is conditioned by several biological and anatomical factors, such as nodal status, tumor size, grade, proliferation index (i.e., Ki67) and others. Even in the era of genomic prediction of outcome, nodal status continues to be one of the strongest prognostic factors. In fact, international guidelines do not recommend Oncotype DX or other prognostic genomic platforms intended to avoid chemotherapy in patients with 4 or more positive nodes [9]. Pan et al. have reported that patients with 4 or more involved nodes (including all breast cancer subtypes) have a risk of distant relapse of 36% at 10 years and 52% at 20 years of follow-up. Patients with 1 to 3 positive nodes have a better long-term prognosis but still have a risk of relapse of 19% at 10 years and 31% at 20 years [10]. In patients with 1 to 3 involved nodes, other additional high-risk factors, such as Oncotype DX recurrence score, grade, tumor size and Ki67 may also be relevant. In the monarchE trial, comparing standard adjuvant endocrine therapy to endocrine therapy plus abemaciclib in EBC patients with high-risk of relapse, the eligibility criteria included patients with 4 or more positive nodes or patients with 1 to 3 involved nodes plus an additional bad prognostic factor (tumor size ≥ 5 cm, grade 3 or centrally determined Ki67 ≥ 20%) [2]. Oncotype DX recurrence score was not taken into consideration in the monarchE trial, and this is one of its weaknesses since postmenopausal patients with 1–3 positive nodes and low to intermediate recurrence score are currently not considered at high-risk of relapse when treated with adjuvant ET alone.

Based on the positive results of monarchE trial (currently with a median follow-up of 42 months), abemaciclib have been approved by FDA, EMA and other medicine agencies for the adjuvant treatment of HR-positive and HER2-negative high-risk breast cancer. Since the report by Pan et al. did not exclude patients with HER2-positive disease, our study aimed to estimate the 10-year outcomes of patients fulfilling the eligibility criteria of monarchE trial. The estimation of the long-term outcomes of patients at high-risk of relapse could be relevant in order to discuss the use of abemaciclib with the patients.

Our study found that the 5 and 10-year risk of distant relapse were 20.1% and 31.4% (15.6% and 24.8% in patients with 1–3 N + and 22.7% and 35.3% with ≥ 4 N +), respectively. Pan et al. provided similar figures for patients with 4 or more N + at these cut-points (22% and 36%) [10]. In the last follow-up of the monarchE trial, the 2- and 4- years iDFS rates in the control group were 89.9% and 79.4% respectively, very similar to the figures in our series (90.4% and 79.3% at 2 and 4 years) [3]. These data suggest that the long-term prognosis of our series can be extrapolated to the control group of the monarchE trial.

A recent study using SEER data from 1975 to 2016 found a 5-year mortality rate of 16.5% for the population of patients eligible for monarchE [11] (versus 11% in our analysis and 12% in the dataset by Pan et al. [10]).

In our study, Ki67 was a prognostic factor of distant relapse rate at 5 years (17.6% y 25.9%) and 10 years (28% y 35.5%) for Ki67 < 20% and ≥ 20% respectively. In the EBCTCG analysis by Pan et al. [10] Ki67 staining was an important independent prognostic factor during the first 5 years but was of only moderate relevance thereafter. However, these two studies used local determination of Ki67, rather than standardized, centralized determinations. The monarchE trial, on the contrary, tested the untreated tumors centrally by means of an investigational Ki67 immunochemistry assay developed by Agilent Technologies (formerly Dako; Santa Clara, CA, USA) and found that Ki67 had a significant prognostic value for distant-relapse free survival of Ki67 after around 2.5 years of median follow-up [4].

In our study, 16.2% of unselected patients from the El Alamo registry, a hospital-based repository including unselected breast cancer patients from 40 Spanish hospitals from 2002 to 2005, would be eligible for abemaciclib according to the EMA approval. A more recent report from Dana-Farber Brigham Cancer Center (2016–2021) found that 11.1% (499/4,496 of consecutive patients with HR-positive and HER2-negative EBC would be eligible for adjuvant abemaciclib [12]. In the previously mentioned SEER analysis, the percentage of patients similar to those included in the monarchE study was of 12% [12]. According to these estimations, a significant proportion of luminal EBC patients (11–16%) would be eligible for adjuvant abemaciclib therapy.

As a limitation, we must admit that the duration of ET used in the patients in our study is probably not the same as that currently used for high-risk patients in routine clinical practice. Also, the administration of LHRH analogs in our series was only used in 15.1% of the premenopausal patients versus half of them in the monarchE study. This could mean that our long-term results may be somewhat worse than those of the control arm population of the monarchE study.

In conclusion, our study shows that the prognosis of patients eligible for the monarchE trial and treated with conventional chemotherapy and ET is dismal, with more than 40% of patients showing an iDFS event and 30% of deaths at 10 years. Looking at the pattern of relapse of patients, with a peak at years 1 till 3 but a plateau from year 4–10, we can speculate that the ideal duration of abemaciclib could be of more than 2 years. These figures could help in the decision-making process for the selection of adjuvant therapy for high-risk EBC patients.

## Supplementary Information

Below is the link to the electronic supplementary material.

Supplementary file1 (DOCX 490 KB)

## Data Availability

The studies from which the patients were selected were completed and closed, except Alamo IV registry which is in data cleaning process. The datasets generated during and/or analyzed during the current study are available from the corresponding author on reasonable request.
