# Supplementary material for: Long-term outcomes of high-risk HR-positive and HER2-negative early breast cancer patients from GEICAM adjuvant studies and El Álamo IV registry
Source: Breast Cancer Res Treat. 2023 Jun 20;201(2):151–9. doi: 10.1007/s10549-023-07002-1 (PMC10361852; doi:10.1007/s10549-023-07002-1)
Supplement: Supplementary file 1 — Supplementary file1 (DOCX 490 KB) [file 10549_2023_7002_MOESM1_ESM.docx]

**Supplementary Table 1.** **Invasive Disease-free Survival (iDFS) and Distant Disease-free Survival (dDFS) First Event Description.**

|  | **Total** |
| --- | --- |
| **Invasive Disease-Free Survival, n (%)** | **740** |
| Local-Regional Invasive Recurrence | 58 (7.8%) |
| Local-Regional + Contralateral Breast Cancer + Distant Recurrence | 2 (0.3%) |
| Local-Regional + Distant Recurrence | 20 (2.7%) |
| Second Primary Breast Cancer | 10 (1.4%) |
| Second Primary Breast Cancer + Contralateral Breast Cancer | 1 (0.1%) |
| Contralateral Breast Cancer | 35 (4.7%) |
| Contralateral Breast Cancer + Second Primary Invasive Cancer (non-breast) | 1 (0.1%) |
| Distant Recurrence | 484 (65.4%) |
| Distant Recurrence + Second Primary Invasive Cancer (non-breast) | 2 (0.3%) |
| Second Primary Invasive Cancer (non-breast) | 72 (9.7%) |
| Death | 55 (7.4%) |
| **Distant Disease-Free Survival**†**, n (%)** | **690** |
| Distant Recurrence | 540 (78.3%) |
| Distant Recurrence + Second Primary Invasive Cancer (non-breast) | 2 (0.3%) |
| Second Primary Invasive Cancer (non-breast) | 77 (11.2%) |
| Death | 71 (10.3%) |

† Distant Disease-Free Survival was defined as time from adjuvant ET initiation to the first date of diagnosis of any of the following events: distant BC relapse, second primary invasive cancer non-BC and death due to any cause.

**Supplementary Table 2.** **Yearly Invasive Recurrence rate (yIRR), Yearly Distant Recurrence Rate (yDRR) and Yearly Death Rate (yDR) in years 1 to 10, overall population, by number of positive axillary lymph nodes (1-3 N+ and ≥ 4 N+) and by Ki67 levels (< 20% and ≥ 20%).**

|  | **Global (n=1617 pts)** | | | | | | | | | | | | |
| --- | --- | --- | --- | --- | --- | --- | --- | --- | --- | --- | --- | --- | --- |
| **Years** | **1** | **2** | **3** | **4** | **5** | **Cumulative at 5 years** | **6** | **7** | **8** | **9** | **10** | **Cumulative at 10 years** |  |
| **IRR (%)** | 2.79 | 5.58 | 6.09 | 4.96 | 4.39 | 21.44% | 3.93 | 4.13 | 3.77 | 3.65 | 3.09 | 33.33% |  |
| 1-3 N+ | 3.01 | 4.70 | 3.86 | 3.25 | 3.37 | 16.72% | 3.54 | 2.00 | 2.66 | 3.70 | 1.41 | 26.09% |  |
| ≥ 4 N+ | 2.66 | 6.09 | 7.41 | 6.02 | 5.04 | 24.21% | 4.18 | 5.52 | 4.55 | 3.61 | 4.31 | 37.60% |  |
| Ki67 < 20% | 1.22 | 4.01 | 6.45 | 5.52 | 3.28 | 18.9% | 3.44 | 2.79 | 2.55 | 3.18 | 2.42 | 29.27% |  |
| Ki67 ≥ 20% | 4.39 | 8.84 | 9.23 | 5.08 | 3.57 | 27.19% | 3.85 | 4.14 | 2.21 | 3.13 | 4.27 | 37.72% |  |
| **DRR (%)** | 2.54 | 5.37 | 5.45 | 4.41 | 4.40 | 20.07% | 3.71 | 3.98 | 3.13 | 3.46 | 3.14 | 31.41% |  |
| 1-3 N+ | 2.84 | 4.52 | 3.30 | 3.03 | 3.33 | 15.55% | 3.50 | 2.20 | 2.15 | 3.64 | 1.66 | 24.75% |  |
| ≥ 4 N+ | 2.36 | 5.97 | 6.83 | 5.37 | 5.08 | 22.74% | 4.11 | 5.85 | 3.97 | 3.89 | 4.22 | 35.33% |  |
| Ki67 < 20% | 1.52 | 4.01 | 5.16 | 5.44 | 3.24 | 17.63% | 2.63 | 3.50 | 2.09 | 4.00 | 2.86 | 27.96% |  |
| Ki67 ≥ 20% | 3.51 | 8.76 | 8.12 | 4.97 | 4.65 | 25.88% | 3.80 | 4.08 | 2.17 | 3.08 | 3.36 | 35.53% |  |
| **DR (%)** | 0.56 | 1.69 | 2.63 | 3.37 | 3.44 | 11.05% | 3.61 | 4.47 | 4.59 | 4.61 | 4.14 | 26.51% |  |
| 1-3 N+ | 0.84 | 1.87 | 1.91 | 2.30 | 2.92 | 9.36% | 3.24 | 2.85 | 2.65 | 3.60 | 2.54 | 20.74% |  |
| ≥ 4 N+ | 0.39 | 1.59 | 3.05 | 4.00 | 3.75 | 12.04% | 3.83 | 5.46 | 5.84 | 5.28 | 5.23 | 29.91% |  |
| Ki67 < 20% | 0.30 | 0.91 | 1.85 | 2.52 | 4.21 | 9.42% | 2.04 | 5.24 | 4.15 | 3.23 | 2.99 | 23.71% |  |
| Ki67 ≥ 20% | 1.75 | 2.68 | 3.24 | 4.78 | 6.06 | 17.03% | 7.14 | 3.05 | 5.77 | 5.56 | 3.82 | 34.50% |  |

The table shows yIRR, yDRR and yDR in years 1 to 10 and cumulative at 5 and 10 years in the overall population, by number of positive axillary lymph nodes (1-3 N+ and ≥ 4 N+) and by Ki67 levels (< 20% and ≥ 20%).

**Supplementary Figure 1. Invasive Disease-free Survival (iDFS) from the start of adjuvant ET. A. By type of ET regimen in Overall population; B. By type of ET regimen in patients with 1-3 N+; C. By type of ET regimen in patients with ≥ 4 N+.**

0

1

2

3

4

5

6

7

8

9

10

11

Time (years)

0

10

20

30

40

50

60

70

80

90

100

Invasive Disease-Free Survival Probability (%)

logrank p-value: <0.001

625

619

602

584

569

542

520

482

449

418

345

255

SERM + AI

453

440

420

394

365

345

310

258

218

205

168

117

AI

448

413

356

305

270

243

223

210

189

171

150

114

SERM

**Number of patients at risk**

87.8%

69.2%

78.2%

57.1%

55.2%

41.1%

0

1

2

3

4

5

6

7

8

9

10

11

Time (years)

0

10

20

30

40

50

60

70

80

90

100

Invasive Disease-Free Survival Probability (%)

logrank p-value: <0.001

228

224

219

214

210

201

191

183

172

159

138

92

SERM + AI

167

162

155

149

138

131

119

102

90

83

73

56

AI

180

168

149

135

128

118

109

105

95

90

81

59

SERM

**Number of patients at risk**

67.4%

54.3%

81.2%

63.2%

89.0%

74.0%

0

1

2

3

4

5

6

7

8

9

10

11

Time (years)

0

10

20

30

40

50

60

70

80

90

100

Invasive Disease-Free Survival Probability (%)

logrank p-value: <0.001

397

395

383

370

359

341

329

299

277

259

207

163

SERM + AI

286

278

265

245

227

214

191

156

128

122

95

61

AI

268

245

207

170

142

125

114

105

94

81

69

55

SERM

**Number of patients at risk**

87.1%

66.5%

76.4%

53.3%

47.0%

32.2%

Kaplan-Meier curves for iDFS were represented for: (A) by type of ET regimen in Overall population; (B) by type of ET regimen in patients with 1-3 N+; (C) by type of ET regimen in patients with ≥ 4 N+.

SERM denotes Selective Estrogen Receptor Modulator and AI denotes Aromatase Inhibitor.

**Supplementary Figure 2. Distant Disease-free Survival (dDFS) from the start of adjuvant ET. A. By type of ET regimen in Overall population; B. By type of ET regimen in patients with 1-3 N+; C. By type of ET regimen in patients with ≥ 4 N+.**

0

1

2

3

4

5

6

7

8

9

10

11

Time (years)

0

10

20

30

40

50

60

70

80

90

100

Distant Disease-Free Survival Probability (%)

logrank p-value: <0.001

625

621

606

589

575

552

528

487

457

428

355

265

SERM + AI

453

441

422

398

375

357

321

267

230

211

173

121

AI

447

416

361

318

285

254

237

224

204

184

164

128

SERM

**Number of patients at risk**

58.1%

89.4%

71.1%

44.9%

59.7%

80.8%

0

1

2

3

4

5

6

7

8

9

10

11

Time (years)

0

10

20

30

40

50

60

70

80

90

100

Distant Disease-Free Survival Probability (%)

logrank p-value: <0.001

228

225

221

216

213

205

195

185

176

163

142

96

SERM + AI

167

162

155

151

143

136

124

107

93

85

75

59

AI

179

168

150

139

132

123

115

111

103

98

90

68

SERM

**Number of patients at risk**

90.8%

75.8%

84.2%

71.2%

60.3%

66.2%

0

1

2

3

4

5

6

7

8

9

10

11

Time (years)

0

10

20

30

40

50

60

70

80

90

100

Distant Disease-Free Survival Probability (%)

logrank p-value: <0.001

397

396

385

373

362

347

333

302

281

265

213

169

SERM + AI

286

279

267

247

232

221

197

160

137

126

98

62

AI

268

248

211

179

153

131

122

113

101

86

74

60

SERM

**Number of patients at risk**

88.7%

68.3%

78.9%

55.9%

49.5%

34.7%

Kaplan-Meier curves for dDFS were represented for: (A) by type of ET regimen in Overall population; (B) by type of ET regimen in patients with 1-3 N+; (C) by type of ET regimen in patients with ≥ 4 N+.

SERM denotes Selective Estrogen Receptor Modulator and AI denotes Aromatase Inhibitor.

**Supplementary Figure 3. Overall Survival (OS) from the start of adjuvant ET. A. By type of ET regimen in Overall population; B. By type of ET regimen in patients with 1-3 N+; C. By type of ET regimen in patients with ≥ 4 N+.**

0

1

2

3

4

5

6

7

8

9

10

11

Time (years)

0

10

20

30

40

50

60

70

80

90

100

Overall Survival Probability (%)

logrank p-value: <0.001

626

626

623

616

604

581

559

529

508

479

406

300

SERM + AI

452

448

439

426

408

394

367

311

272

252

214

154

AI

445

437

417

395

373

345

315

293

256

232

199

159

SERM

**Number of patients at risk**

94.4%

81.1%

90.4%

73.8%

80.3%

55.0%

0

1

2

3

4

5

6

7

8

9

10

11

Time (years)

0

10

20

30

40

50

60

70

80

90

100

Overall Survival Probability (%)

logrank p-value: <0.001

229

229

227

225

221

215

204

195

191

184

161

109

SERM + AI

165

163

159

156

152

145

135

120

106

98

87

67

AI

179

174

167

161

155

145

135

131

119

110

99

76

SERM

**Number of patients at risk**

95.2%

85.7%

92.6%

78.6%

84.1%

67.1%

0

1

2

3

4

5

6

7

8

9

10

11

Time (years)

0

10

20

30

40

50

60

70

80

90

100

Overall Survival Probability (%)

logrank p-value: <0.001

397

397

396

391

383

366

355

334

317

295

245

191

SERM + AI

287

285

280

270

256

249

232

191

166

154

127

87

AI

266

263

250

234

218

200

180

162

137

122

100

83

SERM

**Number of patients at risk**

93.9%

78.5%

89.1%

71.1%

77.8%

46.8%

Kaplan-Meier curves for OS were represented for: (A) by type of ET regimen in Overall population; (B) by type of ET regimen in patients with 1-3 N+; (C) by type of ET regimen in patients with ≥ 4 N+.

SERM denotes Selective Estrogen Receptor Modulator and AI denotes Aromatase Inhibitor.
